# Sinomenine in Cardio-Cerebrovascular Diseases: Potential Therapeutic Effects and Pharmacological Evidences

**DOI:** 10.3389/fcvm.2021.749113

**Published:** 2021-10-01

**Authors:** Meng-Wan Zhang, Xiao-Hui Wang, Jing Shi, Jian-Guang Yu

**Affiliations:** Department of Pharmacy, Shanghai Chest Hospital, Shanghai Jiao Tong University, Shanghai, China

**Keywords:** sinomenine, cardio-cerebrovascular diseases, atherosclerosis, ischemia-reperfusion injury, pharmacological activity, therapeutic effect

## Abstract

Cardio-cerebrovascular diseases, as a major cause of health loss all over the world, contribute to an important part of the global burden of disease. A large number of traditional Chinese medicines have been proved effective both clinically and in pharmacological investigations, with the acceleration of the modernization of Chinese medicine. Sinomenine is the main active constituent of sinomenium acutum and has been generally used in therapies of rheumatoid arthritis and neuralgia. Varieties of pharmacological effects of sinomenine in cardio-cerebrovascular system have been discovered recently, suggesting an inspiring application prospect of sinomenine in cardio-cerebrovascular diseases. Sinomenine may retard the progression of atherosclerosis by attenuating endothelial inflammation, regulating immune cells function, and inhibiting the proliferation of vascular smooth muscle cells. Sinomenine also alleviates chronic cardiac allograft rejection relying on its anti-inflammatory and anti-hyperplastic activities and suppresses autoimmune myocarditis by immunosuppression. Prevention of myocardial or cerebral ischemia-reperfusion injury by sinomenine is associated with its modulation of cardiomyocyte death, inflammation, calcium overload, and oxidative stress. The regulatory effects on vasodilation and electrophysiology make sinomenine a promising drug to treat hypertension and arrhythmia. Here, in this review, we will illustrate the pharmacological activities of sinomenine in cardio-cerebrovascular system and elaborate the underlying mechanisms, as well as give an overview of the potential therapeutic roles of sinomenine in cardio-cerebrovascular diseases, trying to provide clues and bases for its clinical usage.

## Introduction

Sinomenine is an alkaloid isolated from the root and stem of *Sinomenium acutum* Rehder et Wilson or *Sinomenium acutum* var. cinereum., and is the main active chemical component of these traditional Chinese medicine which used to treat rheumatism and neuralgia for centuries (1–4). The classic pharmacological activities of sinomenine are anti-inflammation and immunomodulation, contributing to its potent therapeutic effects on rheumatoid arthritis and sciatic neuritis or lumbalgia (1, 2, 5). Since sinomenine is purified in the 1920s, many other pharmacological properties and therapeutic efficacies of this alkaloid have been discovered and investigated, including promotion of histamine release, mild sedative and analgesic effects, treatment of ankylosing spondylitis, and protection against cardio-cerebrovascular diseases, etc. (1–3, 6–10). The diverse functions of sinomenine make it a promising and effective drug in clinical use.

Cardio-cerebrovascular diseases are the leading cause of death and severely threaten the health and living quality of people worldwide (11–13). As the effectiveness of Chinese medicine in clinical treatments has been recognized gradually around the world, investigations on the specific role of sinomenine in cardio-cerebrovascular diseases are increasing, especially inspired by the fact that inflammatory responses and immune activation are involved in many pathological processes in cardio-cerebrovascular diseases, such as atherosclerosis, as well as cerebral and myocardial ischemia-reperfusion injury (IRI) (14–17). Recently, it has been realized that sinomenine treatment could be beneficial to many cardio-cerebrovascular diseases, including atherosclerosis, cerebral or myocardial IRI, cardiac allograft rejection, autoimmune myocarditis, hypertension, and arrhythmia. The underlying mechanisms involve anti-inflammation, immunosuppression, regulation of cell proliferation and apoptosis, inhibition of oxidative stress and calcium overload, vasodilation, and regulation of electrophysiology. In this review, we will systematically summarize and expound the role of sinomenine in cardio-cerebrovascular diseases based on its pharmacological effects and therapeutic potentials, aiming to give enlightenments for clinical applications of sinomenine.

## Sinomenine and Atherosclerosis

**Figure 1:**
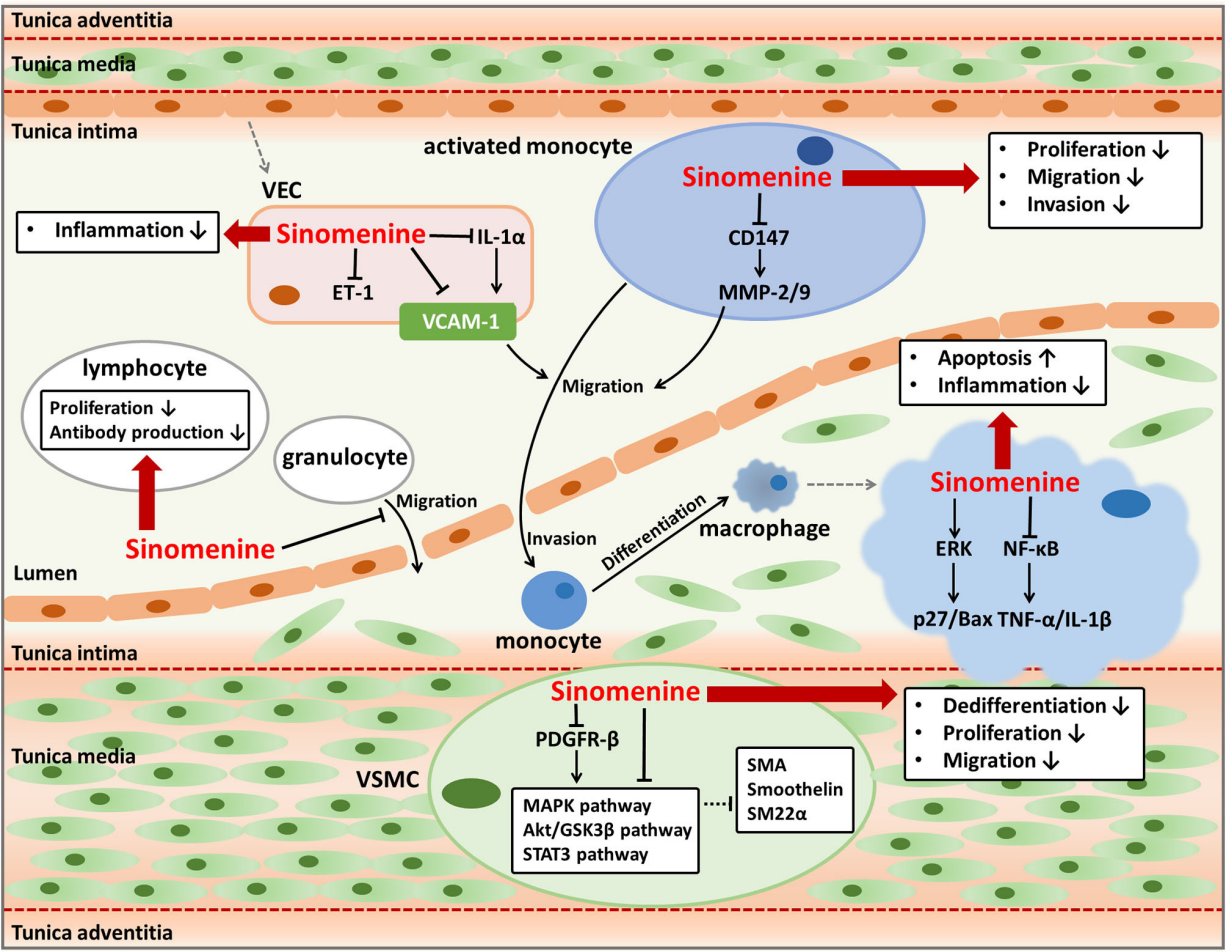
Anti-atherosclerotic effects of sinomenine. Sinomenine could suppress the progression of atherosclerosis by affecting the activities of multiple cells including VECs, monocytes, granulocytes, lymphocytes, macrophages, and VSMCs. Sinomenine attenuates the endothelial inflammation through decreasing pro-inflammatory factors VCAM-1, IL-1α, and ET-1. Sinomenine represses the proliferation, migration, and invasion of monocytes with downregulating CD147 and MMP-2/9. Sinomenine also inhibits the migration of granulocytes, as well as the proliferation and antibody production of lymphocytes. In addition, sinomenine promotes cell apoptosis and decreases the inflammatory response of macrophages through regulating ERK and NF-κB signal pathways respectively, resulting in upregulation of p27 and Bax, and downregulation of TNF-1α and IL-1β. Furthermore, Sinomenine restrains the dedifferentiation, proliferation, and migration of VSMCs depending on inhibition of several signal pathways including MAPK, Akt/GSK3β, STAT3, and PDGFR-β, leading to the increased expression of SMA, Smoothelin, and SM22a.

Atherosclerosis is the major driving factor of coronary artery diseases (15, 18–20). The formation and development of atherosclerotic plaques may cause coronary artery stenosis or blocking and ischemia of myocardium or brain, frequently leading to severe cardio-cerebrovascular diseases such as myocardial infarction, angina, heart failure, and ischemic cerebral stroke (14, 15, 21, 22). Multiple pathological progressions contribute to the initiation and development of atherosclerosis, including lipid metabolic disorder, endothelial dysfunction and inflammation, activation of immune cells, and abnormal cellular activities of vascular smooth muscle cells (VSMCs) (14). Sinomenine is reported to have pharmacological activities such as anti-inflammatory effects on endothelium, immunosuppressive effects on leukocytes, and inhibitory effects on VSMCs proliferation (Figure 1). Besides, drug interactions between sinomenine and other cardiovascular drugs also have been investigated. Therefore, sinomenine is promising to be used for the prevention or treatment of atherosclerosis.

### Suppression on Endothelial Inflammation and Immune Activation

Endothelial inflammation and immune activation are essential pathological processes of atherosclerosis. Long-term chronic inflammation throughout atherosclerosis triggers the assembly and activation of immune cells in the lesions of atherosclerotic plaque, thus contributing to the progress of atherosclerosis (23). Inflammatory responses in vascular endothelial cells (VECs) can prompt the excretion of pro-inflammatory cytokines, activation of multiple signal pathways, and expression of endothelial leukocyte adhesion molecules including E-selectin, intracellular adhesion molecule-1 (ICAM-1), and vascular adhesion molecule-1 (VCAM-1) (14, 24, 25). These cellular activities are responsible for immune responses which play vital roles in the development of atherosclerosis, such as recruitment, invasion, and differentiation of monocytes, and inflammatory activities mediated by macrophages and other leukocytes (15, 23). Therefore, the regulation of endothelial inflammation and immune activation are important ways to treat or prevent atherosclerosis. Sinomenine has strong anti-inflammatory and immunosuppressive activities and hence may have anti-atherogenic effects through regulating endothelial inflammation and function of immune cells that participate in atherosclerosis (26–31).

VCAM-1, expresses and distributes on the activated VECs surface, is a kind of adhesion molecules and can bind to leukocytes and promote their adhesion and trans-endothelial migration (25, 26, 32). Cytokines such as tumor necrosis factor-α (TNF-α) and interleukin (IL)-1 play important roles in stimulating the expression of adhesion molecules including VCAM-1 (33). As VCAM-1 usually indicates the infiltration of leukocytes such as monocyte, macrophage, or lymphocyte, it is thought to be a promising target to detect atherosclerosis and assess the efficacy of anti-atherogenic therapies (25). Sinomenine is found to inhibit VCAM-1 expression induced by TNF-α in VECs (26). Sinomenine downregulates TNF-α and IL-1β through blocking the activity of nuclear factor-kappa B (NF-κB) in macrophages and synoviocytes (27). Sinomenine also inhibits the lipopolysaccharide-induced upregulation of IL-1α in VECs (28). Increased IL-1 has been reported leading to endothelial inflammation by inducing the adhesion and migration of leukocytes to endothelium dependent or independent of VCAM-1 (34). Therefore, the decline of VCAM-1 in VECs caused by sinomenine might be at least in part due to the suppression of TNF-α and IL-1 by regulating the NF-κB signal pathway, which needs further investigation. Besides, Sinomenine has an inhibitory effect on endothelin-1 (ET-1) in VECs (28). ET-1 is a marker of endothelial damages and is involved in inflammation responses (35, 36). As a result, sinomenine can be helpful to alleviate the endothelial inflammation in atherosclerosis by suppressing VCAM-1, IL-1, and ET-1.

The effects of sinomenine on VCAM-1 and pro-inflammatory factors including TNF-α, IL-1, and ET-1 suggest its potent repression on inflammatory and immune responses which facilitate the formation of foam cells and exacerbate the progression of plaque in atherosclerosis. Multiple immune cells participate in different stages of atherosclerosis. Monocytes can be activated and recruited to the damaged endothelium and differentiate into macrophages. Macrophages secret pro-inflammatory factors and ingest lipids, usually the low density lipoproteins, to form the inflammatory foam cells (14, 23). Lymphocytes and granulocytes are also found to be pro-atherogenic and play parts in atherosclerotic plaque formation (15, 37). It is proved that sinomenine can inhibit the proliferation of monocytes and suppress the invasion and migration ability of activated monocytes which differentiate into macrophages (30, 38). The suppression on invasion and migration of monocytes by sinomenine may be related to the reduction of metalloproteinase (MMP)-2 and MMP-9 in the activated monocytes by downregulating the expression of extracellular matrix metalloproteinase inducer (EMMPRIN, CD147) (30). Hence, sinomenine contributes to restraining the leukocytes-endothelial adhesive interactions by suppressing invasion, migration, and differentiation of monocytes. Sinomenine induces the apoptosis of macrophages through activating extracellular signal regulated protein kinase (ERK) to upregulate p27 and pro-apoptotic factor B-cell lymphoma (Bcl)-2-associated x (Bax) (29). p27 is an inhibitor of cyclin E/cyclin-dependent kinase 2 and p27 overexpression may block cell cycle progression and induces apoptosis (39). As a member of the Bcl-2 family, Bax induces the release of inducing factors of apoptosis such as procaspase-9 and cytochrome c (40). Sinomenine also decreases the pro-inflammatory factors production of macrophages including TNF-α, IL-1, and prostaglandin E_2_ (41–44). The role of sinomenine on macrophages may reduce the formation of inflammatory foam cells and atherosclerotic plaques. Besides the impacts on monocytes and macrophages, sinomenine also inhibits proliferation of lymphocytes from mouse spleen, antibody production by B cells, and transmigration of granulocytes across the IL-1β activated human umbilical vein endothelial cells monolayer, conducing to alleviate the progression of atherosclerosis (31, 38, 45). Taken together, sinomenine may suppress proliferation, invasion, migration, and differentiation of monocytes, increase apoptosis of macrophages, and modulate functions of lymphocytes and granulocytes, which benefit for mitigating plaque formation and progression in atherosclerosis.

### Inhibition of VSMCs Proliferation

VSMCs are abundant in the arterial wall and play important parts in atherosclerosis by promoting vascular remodeling, neointima formation, and plaque stability (14, 46, 47). The phenotype switching of VSMCs from contractile to synthetic, characterized by enhanced proliferation and migration abilities and reduced apoptosis of VSMCs, can accelerate atherosclerosis progression (14, 46). Dedifferentiated VSMCs can migrate from the media to the intima of the vessel wall and proliferate rapidly, with higher expression of extracellular matrix components, extracellular matrix-remodeling enzymes, and pro-inflammatory cytokines (48). As the phenotype switching of VSMCs is a reversible process, the regulation on phenotype switching could be developed into a method used for preventing or retarding the progress of atherosclerosis (46).

Sinomenine has an influence on phenotype switching of VSMCs by inhibiting the dedifferentiation, proliferation, and migration of rat VSMCs induced by platelet-derived growth factor-BB. The reversed dedifferentiation of VSMCs by sinomenine is proved by upregulating multiple smooth muscle-specific contractile genes such as smooth muscle α-actin (SMA), smoothelin, and smooth muscle 22α (SM22α). As a result, sinomenine decreases the neointimal formation after carotid artery injury *in vivo*, represented by the reduced intimal area and intima-to-media ratio. The effects of sinomenine on VSMCs phenotype modulation may be due to its inhibition on mitogen-activated protein kinase (MAPK), protein kinase B (Akt)/glycogen synthase kinase 3β (GSK3β), signal transducer and activator of transcription 3 (STAT3), and platelet-derived growth factor receptor-β (PDGFR-β) pathways (21). The phosphorylation of ERK1/2 and p38 in MAPK signal pathway promotes platelet-derived growth factor-BB induced VSMCs dedifferentiation (49–51). The Akt/GSK3β signal pathway modulates VSMCs phenotype to increase its dedifferentiation and is implicated in cell proliferation and migration (49, 52, 53). STAT3 regulates cell growth and differentiation and its phosphorylation is usually in response to acute vascular injury and induces neointimal hyperplasia (54–56). Sinomenine might suppress MAPK, Akt/GSK3β, and STAT3 signal pathways directly or by inhibiting the phosphorylation of their upstream regulator PDGFR-β which can be activated by platelet-derived growth factor-BB and cause the activation of its downstream signal pathways (57, 58). Relying on its effects on phenotype switching of VSMCs, sinomenine could be used to treat vascular proliferative diseases including atherosclerosis and restenosis after percutaneous coronary intervention or vein graft (59, 60).

### Interaction of Sinomenine with Statins

Statins are commonly used to alleviate atherosclerosis and can lower the cardiovascular mortality and the risk of cardiovascular events in patients with coronary artery diseases (61, 62). Statins are usually prescribed for long-term use and combined with other drugs, resulting from the complicated and diverse conditions in cardiovascular diseases. Consequently, drug interactions between statins and other drugs of clinical use have become a special concern for the sake of ensuring the safety and efficacy of medications in these patients (63, 64).

Sinomenine has been reported to have drug interaction with statins, as statins can influence the metabolism of sinomenine in liver and lead to the change of pharmacokinetic parameters of sinomenine. Sinomenine can be metabolized in rat liver microsomes, catalyzed by enzymes CYP3A1/2 and CYP2D1 which are homologous with CYP3A4 and CYP2D6 of human. The inhibitory or inductive effects on sinomenine metabolism by statins are dependent on the dosage and administration period. Single dose of simvastatin or lovastatin could inhibit the liver metabolism of sinomenine, resulting in increased plasma concentration and decreased clearance rates of sinomenine (65). The underlying mechanism could be that simvastatin and lovastatin, as well as their metabolites, are the substrates of CYP3A (66–69). Thus, co-administration of statins may competitively inhibit the metabolism of sinomenine by CYP3A1/2 in rats after a single administration. It also suggests that co-administration of sinomenine might influence the concentration of statins in turn, as well as other drugs catalyzed by CYP3A. However, multiple doses of simvastatin reduce the plasma concentration of sinomenine. The upregulation of CYP3A1/2 at the transcriptional and translational levels by long-term co-administration with simvastatin may account for this opposite effect on sinomenine (65). Upregulation of CYP3A1/2 after long-term co-administration of sinomenine and simvastatin might result from the compensatory mechanism since the two drugs compete for CYP3A1/2 leading to insufficiency of enzymes. Drug interactions between sinomenine and statins give us a hint about how to ensure the effectiveness and safety of both drugs, as well as other drugs catalyzed by CYP3A, in clinical use based on the regulation of dosage and administration period of medication.

## Sinomenine and Ischemic Cardio-Cerebrovascular Diseases

**Figure 2:**
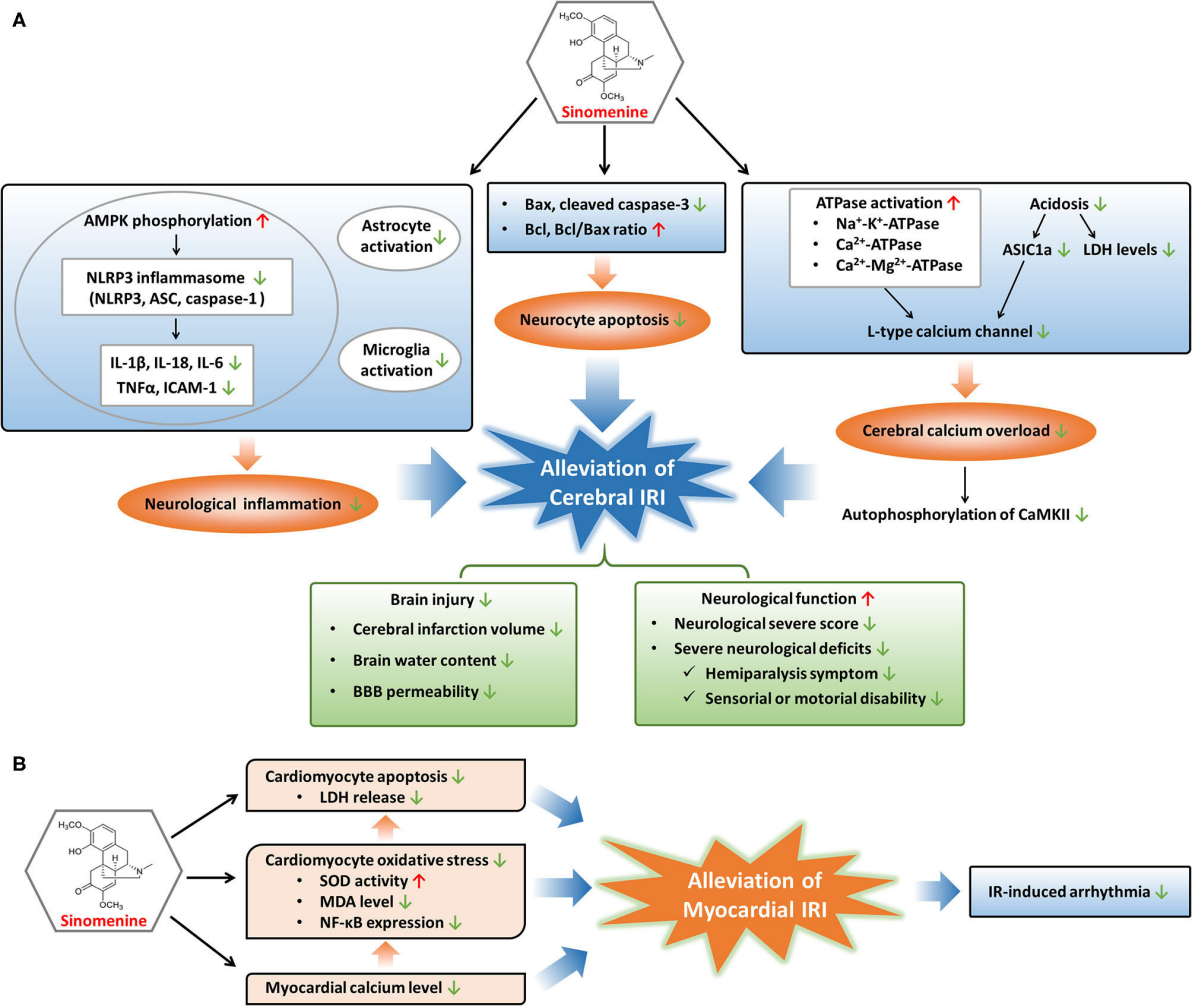
Modulatory mechanisms and protective effects of sinomenine in cerebral and myocardial IRI. **(A)** Sinomenine may alleviate cerebral IRI through inhibition of neurological inflammation, neurocyte apoptosis, and calcium overload in the cerebrum, resulting in the amelioration of brain injury and improvement of neurological function. **(B)** Sinomenine could mitigate myocardial IRI by suppressing the apoptosis and oxidative stress of cardiomyocytes, as well as lowering myocardial calcium level, further leading to the reduction of IR-induced arrhythmia. Red upward arrows represent stimulation by sinomenine, and green downward arrows represent inhibition by sinomenine.

Ischemic cardio-cerebrovascular diseases, typically referring to myocardial infarction and ischemic stroke, are major cause of death and disability globally. IRI is a phenomenon that occurs after the restoration of blood flow in ischemic tissues and is associated with many severe cardio-cerebrovascular diseases (16, 70). IRI is characterized by functional and structural alterations with cellular destruction and dysfunction in ischemic tissues after reperfusion (17). Multiple factors such as oxidative stress, inflammatory and immune response, calcium overload, and dysfunction of mitochondria contribute to the pathogenesis and development of IRI (16, 17, 71). It is urgent to find suitable drugs as the treatment for IRI remains lacking (72, 73). Sinomenine has been reported protective for IRI of the myocardium and brain depending on its regulatory effects on cell death, inflammation, calcium overload, and oxidative stress (Figure 2), which could be a promising drug to treat or prevent IRI in the cardio-cerebrovascular system (10, 74–77).

### Prevention of Cerebral IRI

Cerebral IRI is an important part of brain injuries caused by ischemic stroke with high recurrence and disability rates worldwide (77). Cerebral IRI could lead to cerebral edema, brain hemorrhage, neurocyte death, neurological dysfunction, and poor prognosis such as disability (78–80). As a consequence, effective drugs to prevent or treat cerebral IRI can be helpful to recover neurological function and improve the prognosis of ischemic stroke. Targeting several pathological processes such as cell apoptosis, inflammatory response, and calcium overload may be beneficial to relieve ischemia-reperfusion (IR)-caused brain damages (81–85). Sinomenine, with the ability to cross the blood-brain barrier (BBB), could prevent and alleviate cerebral IRI by inhibiting apoptotic gene activation and the NOD-like receptor family pyrin domain containing 3 (NLRP3) inflammasome-mediated inflammation, as well as attenuating calcium overload through regulating acidosis and energy metabolism, resulting in reverse of neurological functional damages (10, 76, 77, 86–88).

Apoptosis is crucial to the pathogenesis of IRI (89, 90). Both the extrinsic and intrinsic pathways of apoptosis are activated in IRI. The extrinsic pathway functions by recruiting caspase-8 to cleave caspase-3, and the intrinsic pathway leads to upregulation of pro-apoptotic Bax and downregulation of anti-apoptotic Bcl (16, 91). Sinomenine may reduce the neurocytes death induced by oxygen glucose deprivation-reperfusion or middle cerebral artery occlusion models through inhibiting apoptosis, since Bax and cleaved caspase-3 are reduced while Bcl and Bcl/Bax ratio are elevated during treatment of cerebral IRI with sinomenine (76, 87).

Besides suppression of apoptosis, sinomenine protects the cerebrum from IRI through decreasing inflammatory responses, especially NLRP3 inflammasome-mediated inflammation (87). Inflammatory response in brain is an important inducer of cerebral IRI, involving NLRP3 inflammasome activation, pro-inflammatory factors production, gliacyte activation, and immune cells involvement, etc. (17, 92, 93). NLRP3 inflammasome, consisting of NLRP3, apoptosis-associated speck-like protein containing a caspase recruitment domain (ASC), and caspase-1, is a classic and well-characterized inflammasome that can sense and respond to neurological injuries such as cerebral IRI (94–96). Activation and assembly of the NLRP3 inflammasome promote the release of pro-inflammatory cytokines including IL-1β, IL-18, and TNF-α (94, 97, 98). Adenosine 5'-monophosphate-activated protein kinase (AMPK) is an upstream regulator of NLRP3 inflammasome and its inactivation leads to excessive NLRP3 inflammasome activation (99, 100). Sinomenine downregulates IR-induced elevation of NLRP3, ASC, and caspase-1 in cerebral tissues through promoting AMPK phosphorylation (87). Elevation of pro-inflammatory factors, including IL-1β, IL-18, IL-6, TNF-α, and ICAM-1, in cerebral tissues during IR are alleviated through sinomenine treatment, at least in part due to its regulation on NLRP3 inflammasome (86, 87). In addition, the suppressive effects of sinomenine on brain inflammatory responses induced by IR are also proved by inhibition of astrocyte and microglia activation which are vital for the initiation and progression of neurological inflammation following cerebral IR (87).

During cerebral IR, intracellular levels of calcium in neurocytes are elevated resulting from ischemia or hypoxia-induced insufficiency of energy supply, which could harm the cells by increasing production of reactive oxygen species, destructing cell membrane, and causing mitochondrial dysfunction, and eventually lead to cell death (16). Low levels of ATP cause the inactivation of several ATPases including Na^+^-K^+^-ATPase, Ca^2+^-ATPase, and Ca^2+^-Mg^2+^-ATPase, leading to membrane depolarization and activation of voltage-gated calcium channels (VGCCs) which mediate the pumping of calcium into neurocytes (16, 17, 86). Another ion channel mediating the entrance of calcium into neurocytes is Ca^2+^-permeable acid-sensing ion channel 1a (ASIC1a) and its activation is mainly responsible for acidosis-mediated injuries of neurons (101, 102). Since acidosis is a common feature of ischemia-induced brain injuries, ASIC1a is activated during cerebral IR, which increases the uptake of calcium into neurocytes. Calcium overload induced by cerebral IR promotes the autophosphorylation of calcium/calmodulin dependent protein kinase II (CaMKII), a protein kinase regulating many calcium signal-mediated events, and the excessive activation of CaMKII contributes to ischemic brain injuries (103–105). Sinomenine blocks the calcium overload in brain induced by IR-related membrane depolarization and acidosis, through the regulation of ATPases, VGCCs, and ASIC1a (76, 86). Sinomenine improves the energy metabolism of the ischemic cortex by elevating Na^+^-K^+^-ATPase, Ca^2+^-ATPase, and Ca^2+^-Mg^2+^-ATPase levels which are reduced in IR (86). L-type calcium channel, one type of VGCCs, is suppressed by sinomenine, resulting in the reduction of calcium currents into neurocytes (76). The inhibition of L-type calcium channel might result from a direct impact of sinomenine or its indirect effect through enhancement of ATPase activation, as the increased ATPase levels may reduce the membrane depolarization and thus suppress VGCCs. Sinomenine also declines the elevated lactate dehydrogenase (LDH) levels in neurocytes and ischemic cortex induced by IR, as well as increased ASIC1a expression and ASIC1a-mediated calcium uptake induced by extracellular pH reduction, suggesting the alleviation of acidosis in the brain by sinomenine (76, 86). In addition, the suppressive effects of sinomenine on calcium overload induced by cerebral IR are demonstrated by its inhibition on cortical autophosphorylation of CaMKII which responds to upregulation of intercellular calcium concentration (76).

Treatment with sinomenine could contribute to attenuation of brain injury and improvement of neurological function (76, 86, 87). The infarct size can predict long-term adverse events in patients suffering IRI and has been used as an indicator of IRI (72, 73). Sinomenine reduces cerebral infarction volume, brain water content, and BBB permeability induced by middle cerebral artery occlusion. The attenuation of these brain injuries induced by IRI leads to elevation of body weights and better performance on neurological function in middle cerebral artery occlusion-treated animals, including the decrease of neurological severe score (NSS), alleviation of severe neurological deficits such as hemiparalysis symptoms and sensorial or motorial disability (76, 86, 87).

### Prevention of Myocardial IRI

Myocardial IRI is a common pathological process in acute coronary artery diseases after restoring blood flow to the ischemic myocardium with percutaneous coronary intervention (71). Myocardial IRI may aggravate impairment of cardiac function and lead to complications of acute coronary artery diseases such as reperfusion arrhythmia (106, 107). Myocardial IRI often manifests as cardiomyocyte damages including apoptosis, pyroptosis, oxidative injury, and calcium concentration elevation, etc. (71, 89, 90, 108–110). Sinomenine could alleviate myocardial IRI by decreasing calcium concentration, oxidative stress, and apoptosis of cardiomyocytes, and also reduces IR-induced arrhythmia (74, 75).

Similar to cerebral IR, calcium concentration in cardiomyocyte augments during the process of cardiac IR. After reperfusion to the ischemic myocardium, the sudden restoration of blood flow together with increased calcium level can trigger oxidative stress with reduced activity of antioxidases, generation of reactive oxygen species and lipid metabolites, finally inducing cell damages and death (16, 17, 71, 93). Sinomenine reduces the elevated calcium contents in the myocardium induced by IR and inhibits oxidative stress in cardiomyocytes through increasing superoxide dismutase (SOD) activity and decreasing lipid peroxidation metabolite malonaldehyde (MDA) (74, 75). Sinomenine also downregulates the expression of NF-κB in cardiomyocytes stimulated by oxidative stress (75). NF-κB, as a redox-sensitive transcription factor, responds to oxidative stress rapidly and plays significant roles in IRI. Activated NF-κB induces the transcription of target genes, promoting multiple pathogenic activities involved in IRI, such as apoptosis and inflammation, and the inhibitors of NF-κB have been proved to reduce IRI (16, 75, 111). Therefore, sinomenine might protect the myocardium and cardiomyocytes from IRI relying on its inhibitory effects on calcium overload, oxidative stress, and NF-κB function.

Cell death in the myocardium induced by IR is a vital cause of cardiac IRI (16, 71). Apoptosis of cardiomyocytes can be evoked due to IR-induced hypoxia and production of reactive oxygen species, and restraint of apoptosis might be a treatment strategy for myocardial IRI (16, 89, 90). Sinomenine suppresses the apoptosis of cardiomyocytes induced by oxidative stress and decreases the LDH release which is positively related to the degree of cell damage and death, suggesting the potential therapeutic role of sinomenine in cardiac IRI (75). Recently, pyroptosis has been found to promote the death of cardiomyocytes and enlarge myocardial infarct area in IR (110). Pyroptosis is a process of programmed cell death that can be induced by NLRP3 inflammasome-mediated activation of caspase (86, 112). Cardiac IRI can be alleviated by inhibition of pyroptosis with suppressing NLRP3 inflammasome including downregulation of NLRP3 and caspase-1 in cardiomyocytes (113). Since sinomenine can restrain the activation of NLRP3 inflammasome with the decrease of NLRP3, ASC, and caspase-1, it is probable to alleviate cardiac IRI by reducing pyroptosis of cardiomyocytes through inactivating NLRP3 inflammasome (87, 114).

In addition, sinomenine is reported to prevent IR-induced arrhythmia in isolated hearts including decreasing the incidence of ventricular extrasystole, ventricular tachycardia, and ventricular fibrillation, shortening the duration of ventricular fibrillation, and prolonging the incubation of ventricular fibrillation (74). In conclusion, sinomenine may protect the myocardium against IRI through its regulation on calcium overload, oxidative stress, apoptosis, and pyroptosis probably, as well as reduction of IR-induced arrhythmia.

## Sinomenine and Immuno-Related Cardiovascular Diseases

Sinomenine has been demonstrated to possess immunosuppressive and anti-inflammatory activities, including regulating the activation, proliferation, and differentiation of lymphocytes, the differentiation and function of dendritic cells (DCs), and production of pro-inflammatory factors, etc. (1). Such effects make sinomenine suitable for therapy of immune-related disorders such as rheumatoid arthritis, hepatitis, colitis, and allograft rejection (41, 44, 115, 116). Excessive immune responses in cardiac tissue cause several immuno-related cardiovascular diseases, while sinomenine may protect the cardiovascular system from immune response-mediated injury including cardiac allograft rejection and autoimmune myocarditis (116, 117).

### Prevention of Cardiac Allograft Rejection

Cardiac graft is an important therapy to rescue patients suffering severe heart failure. Chronic rejection (CR) with graft vasculopathy is a major cause of cardiac graft failure (118–120). Myocardial fibrosis, perivascular and interstitial inflammatory infiltration mediated by immune cells, and narrowing or occlusion of the graft vasculature due to hyperplasia of vascular intima and VSMCs make contributions to CR of cardiac allograft (118, 119). Besides, the upregulation of vascular endothelial growth factor, basic fibroblast growth factor, and ET-1, which are generated from a variety of cell types such as VSMCs and macrophages, could be stimulated by T cell/B cell-driven immune responses and promote the development of CR in multiple transplant organs (121–123). Sinomenine has been reported to exhibit anti-inflammatory, immunomodulatory, and anti-hyperplastic effects in vessels, suggesting that it may also have therapeutic effects on vasculopathy-related CR in cardiac allograft (14, 15, 24, 26, 28, 32). It is found that sinomenine, compared with untreatment or cyclosporin A, causes less severe vasculopathy in a model of cardiac allograft, representing as lower vasculopathy score, less luminal narrowing, less proportion of diseased vessels, and less fibrotic alterations, as well as reduced mononuclear cell infiltrates and macrophages proportion in cardiac allografts. Furthermore, the combination of sinomenine and cyclosporin A results in more significant improvements on vasculopathy probably through reducing IgM levels and downregulating vascular endothelial growth factor, basic fibroblast growth factor, and ET-1, indicating that sinomenine may act synergistically with cyclosporin A by enhancing the effects of cyclosporin A on vasculopathy, humoral immune response, and expression of cytokines and tissue growth factors (116). Hence, sinomenine could be effective in treatment of chronic cardiac allograft rejection either alone or in combination with other immunosuppressive drugs, which benefits for patients with heart failure.

### Prevention of Autoimmune Myocarditis

Excessive or abnormal immune response in myocardium induced by infectious or non-infectious factors can trigger myocarditis, leading to severe consequences especially in children and young people (124–126). Autoimmune myocarditis is a kind of non-infectious myocarditis, which could be mediated by the activity of DCs (127). DCs are a type of antigen-presenting cells with a strong capacity to induce primary immune responses and act as an essential regulator in immunity and tolerance balance relying on their activation status (128–130). Inhibition of DCs maturation leads to T cell unresponsiveness and inflammation tolerance, resulting in alleviation of autoimmune diseases including autoimmune myocarditis (129, 130). Sinomenine could suppress the maturation of monocyte-derived DCs, represented by downregulation of their mature parameters such as membrane antigens CD40, CD80, CD83, CD86, and human leukocyte antigen DR, resulting in inhibition of T cells activation and IL-12 expression (117). The immunosuppressive effects of sinomenine on DCs may depend on the downregulation of phosphorylation of inhibitor of NF-κB α and inhibition of the nuclear translocation of RelB, leading to the inactivation of NF-κB pathway which has been proved to regulate the maturation of DCs (117, 131–133). In consequence, it provides evidence that sinomenine might have the potency to treat DCs-mediated autoimmune myocarditis.

## Sinomenine and Other Cardiovascular Diseases

### Sinomenine and Hypertension

Vascular tone regulation is crucial to many cardiovascular diseases, especially to hypertension. Relaxation of the vessels could reduce the blood pressure and workload of heart, which plays essential roles in therapy of hypertension and heart failure (8, 134). Sinomenine could dose-dependently mitigate norepinephrine, phenylephrine, KCl, or phorbol 12, 13-dibutyrate-induced vasoconstrictions of isolated aortal rings through multiple mechanisms (8, 134, 135). The activation of protein kinase C (PKC) in VSMCs is an important inducer of vasoconstriction (134, 135). The facts that sinomenine mitigates vasoconstriction induced by phorbol 12, 13-dibutyrate (a PKC activator) and pretreatment with staurosporine (a PKC inhibitor) attenuates the vasodilative effects of sinomenine suggest that sinomenine may function through suppressing the PKC activity in VSMCs (8, 135). Increased calcium concentration in VSMCs triggers contraction of VSMCs and the blockade of calcium channels has become a common method for vasorelaxation and decompression (136, 137). In addition, the opening of ATP-sensitive K^+^ channel in VSMCs could also lead to a decrease of intercellular calcium concentration (138). Sinomenine alleviates the contraction induced by phenylephrine/KCl and lowers the elevated calcium concentration in VSMCs, and pretreatment of nicardipine (a L-type calcium channel blocker) or glibenclamide (a selective ATP-sensitive K^+^ channel blocker) attenuates vasodilative effects of sinomenine. Accordingly, it is possible that sinomenine reduces the calcium concentration in VSMCs to cause vasorelaxation through both blocking L-type calcium channel and opening ATP-sensitive K^+^ channel (8, 134, 135). Besides, sinomenine may activate β-adrenoceptor in VSMCs to relax the vessels since propranolol (a β-adrenoceptor blocker) attenuates vasodilation induced by sinomenine (8). In addition to acting on VSMCs, endothelial-dependent vasorelaxation plays important parts in the vasodilative effects of sinomenine. Removal of endothelium attenuates the vasorelaxation caused by sinomenine (135). Reduction of the endothelium-derived relaxing factor NO and less release of prostaglandin *I*_2_ from endothelium induced by pretreatments with NG-monomethyl-L-arginine, monoacetate salt (a NO synthesis inhibitor) and indomethacin (a cyclooxygenase inhibitor) also result in attenuated vasodilative effects of sinomenine, indicating that sinomenine may elevate NO and prostaglandin *I*_2_ levels to dilate vessels (8, 135).

To sum up, sinomenine could cause vasorelaxation probably relying on its inhibition of PKC activity and L-type calcium channel, accompanied with the activation of ATP-sensitive K^+^ channel and β-adrenoceptor stimulation in VSMCs, as well as its promotion of endothelial-dependent NO and prostaglandin *I*_2_ synthesis (8, 9, 134, 135). It is found that sinomenine only lowers the systolic blood pressure in spontaneously hypertensive rats while has no impact on the systolic blood pressure in normotensive rats, which might partly result from the increased distribution and/or sensitivity of ATP-sensitive K^+^ channel and augmented Ca^2+^ sensitivity induced by PKC during hypertension (134, 139). Therefore, sinomenine may have the potential for controlling blood pressure clinically. Furthermore, since vasodilation is helpful to reduce the pre- and after-loads of the cardiovascular system, which is essential for the treatment of heart failure, sinomenine also could be a hopeful drug to treat heart failure through executing its vasodilative effects (8, 9, 135).

### Sinomenine and Arrhythmia

Dysrhythmia makes great damages to cardiac function, and severe or untreatable arrhythmia may lead to death clinically (9). It has been reported that sinomenine has cardioprotective effects based on its regulation of cardiac rhythm (8, 9, 74, 140). Sinomenine has an impact on action potential configurations in ventricular cardiomyocytes and papillary muscles, including prolonging action potential duration with increase of repolarization and decreasing action potential amplitude with inhibition of the maximum rate of depolarization, probably resulting from its regulation of several ionic currents. Sinomenine could inhibit the L-type Ca^2+^ current, the delayed rectifier K^+^ current, and the inwardly rectifying K^+^ current (I_K1_) in cardiomyocytes, resulting in the prolonging of the action potential duration. The inhibition of the inwardly rectifying K^+^ current by sinomenine could depolarize the membrane potential, which plays a part in anti-arrhythmic actions. Suppression of action potential amplitude and the maximum rate of depolarization suggest the repressive action of the fast Na^+^ current, a class I anti-arrhythmic action, indicating the inhibitory effects of sinomenine on the conduction velocity and excitability. Besides, the reduced fast Na^+^ current might also cause the decline of cellular calcium concentration, which is essential for the alleviation of arrhythmia. As a consequence, the abnormal action potentials induced by calcium overload are suppressed by sinomenine (8, 140). Arrhythmia caused by picrotoxin or BaCl_2_ could also be recovered into sinus rhythm by sinomenine (9). Furthermore, sinomenine attenuates the arrhythmia following IR, as mentioned in the former parts (74). In summary, sinomenine may be used for the treatment of arrhythmia depending on its electropharmacological effects on the action potential configuration and the ionic channel currents.

## Conclusion

Cardio-cerebrovascular diseases are the major cause of public health problems globally and Chinese medicine has broad prospects for treatment of such diseases. Sinomenine possesses promising protective effects on atherosclerosis, cerebral or myocardial IRI, cardiac allograft rejection, autoimmune myocarditis, hypertension and heart failure, as well as arrhythmia, relying on its diverse pharmacological activities including anti-inflammation, immunosuppression, modulation of cell proliferation and apoptosis, attenuation of oxidative stress and calcium overload, and vasodilatory or electrophysiological function. Further investigations are required to focus on the definite therapeutic roles of sinomenine in clinical situation.

## Author Contributions

M-WZ: conceptualization and writing—original draft. X-HW: literature collection. JS: visualization. J-GY: conceptualization and writing—review and editing. All authors contributed to the article and approved the submitted version.

## Funding

This work was supported by grants from the Nurture projects for basic research of Shanghai Chest Hospital (2020YNJCM08 and 2020YNJCQ01) and the National Natural Science Foundation of China (82173812 and 82104161).

## Conflict of Interest

The authors declare that the research was conducted in the absence of any commercial or financial relationships that could be construed as a potential conflict of interest.

## Publisher's Note

All claims expressed in this article are solely those of the authors and do not necessarily represent those of their affiliated organizations, or those of the publisher, the editors and the reviewers. Any product that may be evaluated in this article, or claim that may be made by its manufacturer, is not guaranteed or endorsed by the publisher.
